# Supplementary material for: Topography of cancer-associated immune cells in human solid tumors
Source: eLife. 2018 Sep 4;7:e36967. doi: 10.7554/eLife.36967 (PMC6133554; doi:10.7554/eLife.36967)
Supplement: Supplementary file 1. — This table lists summary statistics of all relevant clinico-pathological features of the DACHS cohort. [file elife-36967-supp1.docx]

| Number of patients | N=286 |
| --- | --- |
| UICC stage | stage I: N=50 stage II: N=102 stage III: N=93 stage IV: N=41 |
| Primary tumor site | Rectum: N=118  Colon: N=168 |
| Received neoadjuvant therapy | No: N=244  Yes: N=41 |
| Age and Sex | Mean age: 68 years  Median age: 69 years  Sex female: N=123  Sex male: N=163 |
| Grading | G1: N=1  G2: N=167  G3: N=74  G4: N=2 |
| Follow up | Death recorded: N=108  Death was due to cancer: N=73  Median follow up time: 5.26 years |

**Suppl. Table 1: Clinical characterization of the DACHS cohort**
