# Supplementary material for: Topography of cancer-associated immune cells in human solid tumors
Source: eLife. 2018 Sep 4;7:e36967. doi: 10.7554/eLife.36967 (PMC6133554; doi:10.7554/eLife.36967)
Supplement: Supplementary file 2. — In this table, all parameters for cell detection and classification using the open source software QuPath are listed. Two sets of parameters are distinguished: ‘DAB’ (diaminobenzidine), used for blue-brown staining, and ‘Red’, used for blue-red staining in melanoma. OD = optical density. All parameters were used in the pan-cancer cohort unless labeled as ‘DACHS’, in which case they were used in the DACHS cohort. [file elife-36967-supp2.docx]

| Parameter | CD3 DAB | CD8 DAB | CD8 DAB DACHS | Foxp3 DAB | CD163 DAB | | | CD 163 DAB DACHS | | CD68 DAB | PD1 DAB |
| --- | --- | --- | --- | --- | --- | --- | --- | --- | --- | --- | --- |
| Scan magnification | 20x or 40x | 20x or 40x | 20x | 20x or 40x | 20x or 40x | | | 20x | | 20x or 40x | 20x or 40x |
| Stain vectors | QuPath default | QuPath default | QuPath default | QuPath default | QuPath default | | | QuPath default | | QuPath default | QuPath default |
| Detection image | Hematox OD | Hematox OD | Hematox OD | OD sum | Hematox OD | | | OD sum | | Hematox OD | Hematox OD |
| Requested pixel size | 0.5 | 0.5 | 0.5 | 0.5 | 0.5 | | | 0.5 | | 0.5 | 0.5 |
| Background radius | 8 | 8 | 8 | 8 | 8 | | | 8 | | 8 | 8 |
| Median filter radius | 0 | 0 | 0 | 0 | 0 | | | 0 | | 0 | 0 |
| Sigma | 1.5 | 1.5 | 1.5 | 1 | 2 | | | 2 | | 2 | 2 |
| Minimum area | 10 | 10 | 10 | 10 | 25 | | | 25 | | 25 | 15 |
| Maximum area | 100 | 100 | 100 | 100 | 250 | | | 250 | | 250 | 100 |
| Intensity threshold | 0.08 | 0.08 | 0.08 | 0.08 | 0.08 | | | 0.06 | | 0.06 | 0.07 |
| Max. background intensity | 2 | 2 | 2 | 2 | 2 | | | 2 | | 2 | 2 |
| Split by shape | on | on | on | on | on | | | on | | on | on |
| Exclude DAB | off | off | off | off | off | | | off | | off | off |
| Cell expansion | 1.5 | 1.5 | 1.5 | 3 | 3 | | | 3 | | 3 | 3 |
| Include cell nucleus | on | on | on | on | on | | | on | | on | on |
| Smooth boundaries | on | on | on | on | on | | | on | | on | on |
| Make measurements | on | on | on | on | on | | | on | | on | on |
| Score compartment | Nucleus DAB OD mean | Nucleus DAB OD mean | Nucleus DAB OD mean | Nucleus DAB OD mean | Nucleus DAB OD mean | | | Nucleus DAB OD mean | | Nucleus DAB OD mean | Cell DAB OD mean |
| Threshold 1+ | 0.2 | 0.14 | 0.25 | 0.33 | 0.2 | | | 0.4 | | 0.18 | 0.18 |
| single threshold | on | on | on | on | on | | | on | | on | on |
|  |  |  |  |  |  | | |  | |  |  |
| Parameter | CD3 Red | CD8 Red | Foxp3 Red | CD163 Red | | CD68 Red | PD1 Red | |  |  |  |
| Scan magnification | 20x or 40x | 20x or 40x | 20x or 40x | 20x or 40x | | 20x or 40x | 20x or 40x | |  |  |  |
| Stain vectors | pick manually | pick manually | pick manually | pick manually | | pick manually | pick manually | |  |  |  |
| Detection image | Hematox OD | Hematox OD | OD sum | OD sum | | OD sum | Hematox OD | |  |  |  |
| Requested pixel size | 0.5 | 0.5 | 0.5 | 0.5 | | 0.5 | 0.5 | |  |  |  |
| Background radius | 8 | 8 | 8 | 8 | | 8 | 8 | |  |  |  |
| Median filter radius | 0 | 0 | 0 | 0 | | 0 | 0 | |  |  |  |
| Sigma | 2 | 2 | 1 | 2 | | 2 | 1 | |  |  |  |
| Minimum area | 15 | 10 | 10 | 25 | | 25 | 15 | |  |  |  |
| Maximum area | 100 | 100 | 100 | 250 | | 250 | 100 | |  |  |  |
| Intensity threshold | 0.06 | 0.08 | 0.08 | 0.07 | | 0.07 | 0.07 | |  |  |  |
| Max. background intensity | 2 | 2 | 2 | 2 | | 2 | 2 | |  |  |  |
| Split by shape | on | on | on | on | | on | on | |  |  |  |
| Exclude DAB | off | off | off | off | | off | off | |  |  |  |
| Cell expansion | 1.5 | 1.5 | 3 | 3 | | 3 | 3 | |  |  |  |
| Include cell nucleus | on | on | on | on | | on | on | |  |  |  |
| Smooth boundaries | on | on | on | on | | on | on | |  |  |  |
| Make measurements | on | on | on | on | | on | on | |  |  |  |
| Score compartment | Cytoplasm DAB OD mean | Cytoplasm DAB OD mean | Nucleus DAB OD mean | Nucleus DAB OD mean | | Nucleus DAB OD mean | Cell DAB OD mean | |  |  |  |
| Threshold 1+ | 0.2 | 0.33 | 0.45 | 0.58 | | 0.33 | 0.25 | |  |  |  |
| single threshold | on | on | on | on | | on | on | |  |  |  |

**Suppl. Table 2: List of all image analysis parameters.** In this table, all parameters for cell detection and classification using the open source software QuPath are listed. Two sets of parameters are distinguished: “DAB” (diaminobenzidine), used for blue-brown staining, and “Red”, used for blue-red staining in melanoma. OD = optical density. All parameters were used in the pan-cancer cohort unless labeled as “DACHS”, in which case they were used in the DACHS cohort.
