# Supplementary material for: Topography of cancer-associated immune cells in human solid tumors
Source: eLife. 2018 Sep 4;7:e36967. doi: 10.7554/eLife.36967 (PMC6133554; doi:10.7554/eLife.36967)
Supplement: Supplementary file 4. — On the full data set of N = 965 tissue slides from N = 177 patients in 10 tumor types, we calculated the median cell density for each antigen, taking the compartments ‘outer invasive margin’ and ‘tumor core’ into account. These median values were subsequently used as cutoff values for low and high cell densities which were then used to define hot, cold and excluded phenotypes. [file elife-36967-supp4.docx]

| **Antigen** | **Cutoff (cells/mm²)** |
| --- | --- |
| CD3 | 363.07 |
| CD8 | 294.88 |
| Foxp3 | 62.31 |
| PD1 | 5.76 |
| CD68 | 310.38 |
| CD163 | 558.95 |

**Suppl. Table 4: List of all cutoff values for all cell types.** On the full data set of N=965 tissue slides from N=177 patients in 10 tumor types, we calculated the median cell density for each antigen, taking the compartments “outer invasive margin” and “tumor core” into account. These median values were subsequently used as cutoff values for low and high cell densities which were then used to define hot, cold and excluded phenotypes.
