# Supplementary material for: Topography of cancer-associated immune cells in human solid tumors
Source: eLife. 2018 Sep 4;7:e36967. doi: 10.7554/eLife.36967 (PMC6133554; doi:10.7554/eLife.36967)
Supplement: Supplementary file 5. — A multivariable Cox proportional hazard model was fitted to all variables listed in this table. N = 286 CRC patients in the DACHS cohort, number of events = 108, significance codes (sig): *<0.05, **<0.01, ***<0.001. HR = hazard ratio, UICC = Union internationale contre le cancer. [file elife-36967-supp5.docx]

|  | HR | lower .95 | upper .95 | p | sig |
| --- | --- | --- | --- | --- | --- |
| CD163_MARG_500_OUT | 1.00 | 1.00 | 1.00 | 0.806 |  |
| CD163_TU_CORE | 1.00 | 1.00 | 1.00 | 0.588 |  |
| CD8_MARG_500_OUT | 1.00 | 1.00 | 1.00 | 0.532 |  |
| CD8_TU_CORE | 1.00 | 1.00 | 1.00 | 0.781 |  |
| UICC stage (1-4, continuous) | 2.27 | 1.80 | 2.87 | 0.000 | *** |
| Age (years, continuous) | 1.05 | 1.02 | 1.07 | 0.000 | *** |
| Sex | 0.79 | 0.53 | 1.16 | 0.223 |  |

**Suppl. Table 5: Continuous cell densities of CD8+ and CD163+ cells are not significantly associated with overall survival in colorectal cancer.**  A multivariable Cox proportional hazard model was fitted to all variables listed in this table. N=286 CRC patients in the DACHS cohort, number of events = 108, significance codes (sig): * < 0.05, ** < 0.01, *** < 0.001. HR = hazard ratio, UICC = Union internationale contre le cancer.
