# Supplementary material for: Topography of cancer-associated immune cells in human solid tumors
Source: eLife. 2018 Sep 4;7:e36967. doi: 10.7554/eLife.36967 (PMC6133554; doi:10.7554/eLife.36967)
Supplement: Supplementary file 6. — A multivariable Cox proportional hazard model was fitted to all variables listed in this table. N = 286 CRC patients in the DACHS cohort, number of events = 108, significance codes (sig): *<0.05, **<0.01, ***<0.001. HR = hazard ratio, UICC = Union internationale contre le cancer. [file elife-36967-supp6.docx]

|  | HR | lower .95 | upper .95 | p | sig |
| --- | --- | --- | --- | --- | --- |
| CD8-cold, CD163-excl | 1.05 | 0.63 | 1.74 | 0.858 |  |
| CD8-cold, CD163-hot | 0.61 | 0.15 | 2.54 | 0.499 |  |
| CD8-excl, CD163-cold | 0.94 | 0.40 | 2.21 | 0.880 |  |
| CD8-excl, CD163-excl | 1.75 | 1.02 | 2.99 | 0.041 | * |
| CD8-excl, CD163-hot | 2.71 | 1.13 | 6.49 | 0.025 | * |
| CD8-hot, CD163-cold | 0.48 | 0.07 | 3.51 | 0.470 |  |
| CD8-hot, CD163-excl | 0.93 | 0.22 | 3.91 | 0.925 |  |
| CD8-hot, CD163-hot | 1.68 | 0.60 | 4.74 | 0.323 |  |
| UICC stage (1-4, continuous) | 2.27 | 1.80 | 2.86 | 0.000 | *** |
| Age (years, continuous) | 1.04 | 1.02 | 1.06 | 0.000 | *** |
| Sex | 0.76 | 0.51 | 1.12 | 0.161 |  |

**Suppl. Table 6: Bivariate immune phenotype predicts risk of death of any cause.** A multivariable Cox proportional hazard model was fitted to all variables listed in this table. N=286 CRC patients in the DACHS cohort, number of events = 108, significance codes (sig): * < 0.05, ** < 0.01, *** < 0.001. HR = hazard ratio, UICC = Union internationale contre le cancer.
